# Impact of gasoline inhalation on some neurobehavioural characteristics of male rats

**DOI:** 10.1186/1472-6793-9-21

**Published:** 2009-11-24

**Authors:** Amal A Kinawy

**Affiliations:** 1 Psychology department, Faculty of Arts, Cairo University, Egypt

## Abstract

**Background:**

This paper examines closely and compares the potential hazards of inhalation of two types of gasoline (car fuel). The first type is the commonly use leaded gasoline and the second is the unleaded type enriched with oxygenate additives as lead substituent in order to raise the octane number. The impacts of gasoline exposure on Na^+^, K^+^-ATPase, superoxide dismutase (SOD), acetylcholinesterase (AChE), total protein, reduced glutathione (GSH), and lipid peroxidation (TBARS) in the cerebral cortex, and monoamine neurotransmitters dopamine (DA), norepinephrine (NE) and serotonin (5-HT) in the cerebral cortex, hippocampus, cerebellum and hypothalamus were evaluated. The effect of gasoline exposure on the aggressive behaviour tests was also studied.

**Results:**

The present results revealed that gasoline inhalation induced significant fluctuations in the levels of the monoamine neurotransmitters in the studied brain regions. This was concomitant with a decrease in Na^+^, K^+^-ATPase activity and total protein content. Moreover, the group exposed to the unleaded gasoline exhibited an increase in lipid peroxidation and a decrease in AChE and superoxide dismutase activities. These physiological impairments were accompanied with a higher tendency towards aggressive behaviour as a consequence to gasoline inhalation.

**Conclusion:**

It is concluded from the present work that chronic exposure to either the leaded or the unleaded gasoline vapours impaired the levels of monoamine neurotransmitters and other biochemical parameters in different brain areas and modulated several behavioural aspects related to aggression in rats.

## Background

Gasoline is the generic term for petroleum fuel used mainly for internal combustion engines. It is complex, volatile, and highly flammable and contains over 500 saturated or unsaturated hydrocarbons having from 3 to 12 carbon atoms. About 110 million people are exposed to gasoline constituents in the course of refueling at gasoline stations [1]. Major toxic risk comes from breathing exhaust fumes and evaporative and refueling emissions rather than from occasional skin contact from spills [2]. Nonetheless, after gasoline application to skin, a decrease in glutathione concentration, glutathione *S *-transferase activity, and lipid peroxidation was observed in liver and brain [3]. Gasoline-induced neurotoxic effects such as ataxia, tremor, acute or subacute encephalopathic syndrome were ascribable to intentional use (gasoline sniffing) and not to occupational exposure. Unfortunately, gasoline sniffing has become an increasingly rising phenomenon in the poor societies as a means for cheap mood alteration [4].

Leaded gasoline contains a range of organolead compounds, especially tetraethyl lead, are difficult to eliminate from the CNS, and the injuries induced usually result in permanent neurological deficits that cause medical as well as socio-economical problems [5]. Ethylene glycol is another gasoline constituent and a general fuel additive widely used in automobiles for its ability to absorb water and to prevent overheating or freezing. Ingestion of ethylene glycol produces not only central nervous system depression, but also cardiopulmonary complications, acute renal failure, and delayed neurological sequelae [6].

The change from leaded to unleaded gasoline, in fact, represents a transformation of a risk factor for the human population chronically exposed to urban air polluted by automobile smokes [7].

Oxygenates include substances such as methanol, ethanol, Methyl tertiary butyl ether (MTBE), ethyl tertiary butyl ether (ETBE), tertiary butyl alcohol (TBA), and tertiary amyl methyl ether (TAME) are used as antiknock agents in place of lead derivatives and as substitutes for high octane aromatics in fuel [8,9]. Methanol are toxic alcohols can be used as a fuel either alone or as a gasoline additive. Methanol is well absorbed in humans following inhalation, ingestion, or cutaneous exposure; it produces a transient mild depression of the CNS with headache, vertigo, and vomiting. Ethanol induced an increase in the levels of norepinephrine in paternally exposed offspring. Serotonin levels were reduced in the cerebrum, and met-enkephalin levels were increased in all brain regions of offspring from both maternally and paternally exposed rats [10]. Methyl tertiary butyl ether (MTBE) is an aliphatic ether, a volatile, colorless, and inflammable liquid, currently the most widely used oxygenate as an octane enhancer in gasoline blends. In subchronic and chronic toxicity studies, MTBE appears to have low systemic toxicity; the main findings observed in rats were depressant effects on the CNS, typical of similar ethers [11,12]. Tertiary amyl methyl ether (TAME) causes a significant but transient CNS depression akin to, but slightly more severe than, that resulting from MTBE exposure at the same levels [13]. Methylcyclopentadienyl manganese tricarbonyl (MMT) is an effective octane enhancer. The primary combustion products of MMT are Mn phosphate, Mn sulfate, and a Mn phosphate/Mn sulfate mixture. Concerns have been raised that the combustion products of MMT containing Mn could cause neurological symptoms similar to Parkinson's disease in humans, even at low levels of exposure [14,15]. In rats, increase in brain Mn delivery was observed with inhalation exposure following Mn absorption from the pulmonary tract and direct transport of Mn to the central nervous system along the olfactory nerve [16]. High dose manganese selectively targets dopaminergic neurons in the human basal ganglia [17,18]. Another study by Erikson *et al *[19] found that rats had significantly decreased striatal glutathione (GSH) levels following subchronic MnSO4 inhalation.

Alkylbenzenes, including toluene and xylene, occur naturally in small amounts in gasoline blends and standard gasoline formulations. These compounds are mainly absorbed by inhalation causing dysfunction in the CNS. The effects vary from severe neurological disorder (acute inhalation due to sniffing abuse) to deficits in neurobehavioural function in occupationally exposed groups. Neurobehavioural effects deriving from sub-acute exposure to toluene have been investigated in rats [20] and mice [21].

As Egypt is now in the process of transferring into the use of unleaded gasoline, this study employed the two types of gasoline currently used in Egypt and focused on comparing their impact. The study was designed to evaluate the effects of gasoline vapour inhalation on the brain monoamine neurotransmitters, lipid peroxidation, GSH, SOD, Na^+^, K^+^-ATPase and AChE. In addition to these biochemical analyses, the impact of gasoline inhalation on the aggressive behaviour was assessed.

## Methods

### Animals

Male Wister rats weighing (130-160 g) obtained from the institute of Ophthalmic Disease Research were used and housed two per cage. Animals were kept in a controlled environment of 12 h light- dark cycle and 22 ± 2°c room temperature. Food and water were provided *ad libitum*. Animals were maintained at the Faculty of Science, Ain Shams University animal unit where housing, handling, and use of animals were strictly in agreement with the regulations and guidelines set by the Faculty's research ethics committee.

### Gasoline vapour inhalation studies

The dynamic system of exposure to gasoline vapours which closely matches exposure to vapour in open air was applied in this study. The dynamic model of exposure to vapours is preferred to the static system as the latter allows the concentration of gas to build up and increase as time goes by Haschek *et al *[22].

### Experimental design

Forty five male rats were randomly divided into three groups each group contain 15: the first group exposed to the 1/5 LC 50 of leaded gasoline (G_1_:7495 PPM) for six consecutive weeks for 30 minutes in the exposure apparatus described by Ezzat *et al *[23], the second group exposed to the 1/5 LC 50 of unleaded gasoline (G_2_: 7985.6 PPM) for 30 minutes in the same exposure apparatus, whereas the control group was kept in a similar apparatus as the other two groups and was exposed to gasoline-free air flow for 30 minutes also (the control or gasoline-free and the inhaled gasoline were under the same condition and the rats of both groups exposed to one variable). Briefly, the apparatus for gasoline inhalation consists of glass inhalation chamber, and a vaporizer (atomizer). Inside the chamber, a barometer, thermometer and hygrometer were installed in order to continuously register the pressure, temperature and humidity, respectively. The animals were kept in an inhalation chamber and the gasoline was evaporated in a vaporizer and the vapours passed through a rubber tube to the inhalation chamber. By calculating the amount of gasoline vapours was determined as part per million (ppm) according to the following equation **PV = n RT (P = **Pressure inside the chamber, **V **= Volume of gasoline in the space, **n **= Number of gasoline moles, **R **= Equation constant of gasoline, **T **= Temperature inside the chamber, according to Smith *et al *[24]).

The LC 50 (30 minutes) for leaded (G_1_) was found to be 37475 ppm and for unleaded (G_2_) 39928 ppm.

The rats of each group were subjected to aggressive behaviour tests for ten days, through the last ten days of exposure. After the completion of this test, the 15 animals of each group were decapitated and the brain was quickly removed out from each rat. The cerebral cortex was excised, from the level of the pituitary gland to the end of the olfactory lobe, for the determination of lipid peroxidation, GSH, SOD, protein, Na^+^, K^+^-ATPase, the AChE and monoamines. Hippocampus, hypothalamus and cerebellum were also excised and used for the determination of monoamines only. Due to the limitation of the tissues, six samples only were analyzed for each parameter. The analysis of each individual sample was repeated three times and the average was recorded.

### A- Aggressive behaviour evaluations

The animals were housed in pairs with like-sexed littermates with whom they had lived continuously. The following parameters were determined according to Barnett [25]:

▪ Tooth chattering: it goes on while the rat remains immobile, after he has detected the presence of an opponent, and while he approaches the latter.

▪ Threat posture: the back is maximally arched by the attacker, all fore limbs are extended and the flank is turned towards the opponent.

▪ Leaping and biting: the attacker springs in the air and comes down on his opponent repeatedly striking at him by rapidly adducting his extended fore limbs and he also bites the ear or limb or the tail.

▪ Boxing position: longer intervals in which both rats adopt a defensive attitude.

### B- Physiological and biochemical measurements

#### Determination of superoxide dismutase (SOD) activity

Superoxide dismutase (SOD) activity was assayed by the procedure of Niskikimi *et al*. [26]. The assay relies on the ability of the enzyme to inhibit phenazine methosulphate-mediated reaction of nitroblue tetrazolium dye.

#### Determination of reduced glutathione (GSH) content

This method was described by Prins and Loose [27] and depends on the precipitation of protein using tungstate-sulfuric acid solution and the formation of a yellow colour after reacting with 5,5'dithiobis-2-nitrobenzoic acid (DTNB) which is then read at 412 nm.

#### Determination of lipid peroxidation product (malondialdehyde, MDA) in brain tissue homogenate

This method is based on the determination of malondialdehyde (MDA), an end product of lipid peroxidation, which reacts with thiobarbituric acid to yield a pink coloured trimethine complex exhibiting a maximum absorbance at 530-532 nm [28].

### HPLC determination of the brain monoamine concentrations

#### Preparation of the sample

The first step in determination of the brain monoamine by HPLC method involved weighing and homogenization of the tissue in 1/10 weight/volume of 75% aqueous HPLC grade methanol. The homogenate was spun at 3000 r.p.m. for 10 min and the supernatant was used for monoamine determination immediately extracted from the trace elements and lipids by the use of solid phase extraction CHROMABOND column NH2 phase Cat. No. 730031. The sample was then injected directly into an AQUA column 150 54.6 mm 5 μC18, purchased from Phenomenex, USA under the following conditions: mobile phase 97/3 20 Mm potassium phosphate, pH 3.0/methanol, flow rate 1.5 ml/min, UV 270 nm. norepinephrine, dopamine and serotonin were separated after 12 minutes. The resulting chromatogram identified each monoamine position and concentration from the sample as compared to that of the standard, and finally, the determination of the content of each monoamine as μg per gram brain tissue [29].

#### Determination of acetylcholinesterase (AChE) activity

The AChE activity was estimated using Ellman procedure [30] as modified by Gorun *et al*. [31] for the assay of purified preparations of cholinesterases. This procedure is based on the reaction of thiocholine, resulting from enzymatic hydrolysis of acylthiocholines with 5, 5'dithiobis-2-nitrobenzoic acid (DTNB). However, the original Ellman procedure was found inadequate for crude enzymatic preparations due to the interfering reactions of free SH groups of protein with DTNB. These limitations have been overcome by transforming the kinetic Ellman procedure into static one where the cholinesterases were incubated with an acylthiocholine for a specific time interval and the reaction was then stopped and the colour was developed upon the addition of DTNB - Phosphate - ethanol reagent. The colour was then read immediately at 412 nm and the enzyme activity was determined in μM per SH group (μMSH) from a standard curve.

#### Determination of total proteins

The total protein content was determined according to the method described by Lowry *et al*. [32]. This method is based on the reaction between the tryptophan and tyrosine residues of the protein and the phosphomolybdic - phosphotungestic acid reagent (commercially known as Folin Ciocalteu's reagent) in alkaline copper solution. The developed blue colour was then measured colouremetrically at a wave-length of 690 nm.

#### Determination of total adenosine triphosphatase (Na^+^, K^+^-ATPase) activity

The principle of the assay depends on the incubation of adenosine triphosphate with the tissue-containing enzyme and the determination of liberated inorganic phosphorus as modified by El-Aaser and El-Merzabani [33].

### Statistical analysis

The data were subjected to statistical analysis using one way ANOVA. SPSS for Windows software, Release 11.0 (SPSS, Chicago, IL) was used.

## Results

Data in table 1 show that the rats exposed to unleaded gasoline exhibited a decrease in the content of norepinephrine of the cerebral cortex in comparison with the control group. On the other hand, no statistically meaningful change was detected between the leaded and unleaded inhaled groups. Moreover, the content of dopamine was lower than that in the control or leaded gasoline groups. On the other hand, no statistical difference was found between the control and the leaded groups. Serotonin content was not affected in all the three experimental groups when comparison was made between each other.

**Table 1:** Effect of chronic exposure to two types of gasoline vapour on the content of monoamines (μg/g) in the cerebral cortex of male rats.

	Parameter		
**GROUP**	**Norepinephrine**	**Dopamine**	**Serotonin**

Control	0.496 ± 0.045	1.3 ± 0.11	0.46 ± 0.06

Leaded gasoline	0.488 ± 0.054	1.36 ± 0.35	0.47 ± 0.18

Unleaded gasoline	0.428 ± 0.062 a*	0.95 ± 0.06 a* b*	0.498 ± 0.053

Values are expressed as means ± SD

a: significantly different from the control group.

b: significantly different from the leaded gasoline.

Asterisks indicate the level of significance (* P < 0.05)

The number of animals in each experimental group was 6.

In the hypothalamus, data in table 2 reveal that the norepinephrine, dopamine and serotonin in the group exposed to the unleaded gasoline were lower than those of the control group. Moreover, norepinephrine in this group was less than that in the leaded gasoline group and the dopamine was higher than that in the leaded gasoline inhaled group. Exposure to leaded gasoline induced a significant decrease in both dopamine and serotonin in comparison with the control group. Moreover, norepinephrine was unaffected in this group as compared with the corresponding values of the control.

**Table 2:** Effect of chronic exposure to two types of gasoline vapour on the content of monoamines (μg/g) in the hypothalamus of male rats

	Parameter		
**GROUP**	**Norepinephrine**	**Dopamine**	**Serotonin**

Control	0.115 ± 0.031	2.68 ± 0.27	0.822 ± 0.047

Leaded gasoline	0.11 ± 0.013	1.136 ± 0.29 a***	0.50 ± 0.075 a***

Unleaded gasoline	0.076 ± 0.0038 a** b**	1.61 ± 0.28 a*** b*	0.49 ± 0.13 a***

Values are expressed as means ± SD

a: significantly different from the control group.

b: significantly different from the leaded gasoline.

Asterisks indicate the level of significance (* P < 0.05; ** P 0.01; *** P < 0.001.)

The number of animals in each experimental group was 6

Data presented in tables 3 show that the rats exposed to the two types of vapours encountered a significant decrease in the norepinephrine, dopamine and serotonin contents of the hippocampus as compared with the control group. On other hand, no significant changes were found in norepinephrine and serotonin between the leaded or the unleaded gasoline, but dopamine level was lower in the unleaded gasoline than leaded gasoline groups.

**Table 3:** Effect of chronic exposure to two types of gasoline vapour on the content of monoamines (μg/g) in the hippocampus of male rats

	Parameter		
**GROUP**	**Norepinephrine**	**Dopamine**	**Serotonin**

Control	0.54 ± 0.066	1.82 ± 0.125	0.65 ± 0.12

Leaded gasoline	0.23 ± 0.035 a***	1.53 ± 0.29 a*	0.4 ± 0.15 a**

Unleaded gasoline	0.25 ± 0.12 a***	1.02 ± 0.11 a*** b**	0.39 ± 0.077 a**

Values are expressed as means ± SD

a: significantly different from the control group.

b: significantly different from the leaded gasoline.

Asterisks indicate the level of significance (* P < 0.05; ** P 0.01; *** P < 0.001.)

The number of animals in each experimental group was 6.

As shown in table 4, norepinephrine and dopamine levels were not significantly changed in the group inhaled the unleaded gasoline in comparison with the control group, on the other hand, serotonin was lower than the other two groups and dopamine was less than that in the leaded gasoline group. Serotonin content of the cerebellum was lower in the leaded gasoline group than control group, but the dopamine and norepinephrine were elevated above the control level.

**Table 4:** Effect of chronic exposure to two types of gasoline vapour on the content of monoamines (μg/g) in the cerebellum of male rats

	Parameter		
**GROUP**	**Norepinephrine**	**Dopamine**	**Serotonin**

Control	0.376 ± 0.07	1.45 ± 0.12	0.80 ± 0.115

Leaded gasoline	0.47 ± 0.086 a*	1.71 ± 0.14 a*	0.60 ± 0.043 a**

Unleaded gasoline	0.41 ± 0.036	1.35 ± 0.24 b**	0.385 ± 0.057 a*** b**

Values are expressed as means ± SD

a: significantly different from the control group.

b: significantly different from the leaded gasoline.

Asterisks indicate the level of significance (* P < 0.05; ** P 0.01; *** P < 0.001.)

The number of animals in each experimental group was 6.

Data in table 5 reveal that Na^+^, K^+^-ATPase activity and total protein content in the two groups exposed to gasoline were lower than the control, although no significant difference was found between each other. The rats exposed to the unleaded gasoline had a significantly lower acetylcholinesterase activity as compared with the control or leaded group. No statistical changes were detected in the acetylcholinesterase activity of the leaded group as compared with the control.

**Table 5:** Effect of chronic exposure to two types of gasoline vapour on the acetylcholinesterase (AChE) activity, Na^+^, K^+^-ATPase activity and total protein of cerebral cortex in male rats

	Parameter		
**GROUP**	**AChE**	**Na^+^, K^+^-ATPase**	**Protein**
	**μmol SH/g/min**	**μmol/pi-min/g wet tissue**	**g/100 g tissue**

Control	17.5 ± 2.24	16.34 ± 1.51	6.63 ± 0.26

Leaded gasoline	16.25 ± 2.2	13.5 ± 1.63 a*	5.78 ± 0.39a**

Unleaded gasoline	12.5 ± 2.62 a** b*	14.21 ± 1.92 a*	6.05 ± 0.38 a*

Values are expressed as means ± SD

a: significantly different from the control group.

b: significantly different from the leaded gasoline.

Asterisks indicate the level of significance (* P < 0.05; ** P 0.01.)

The number of animals in each experimental group was 6.

Table 6 shows that the rats exposed to the unleaded gasoline had a significant increase in lipid peroxidation in comparison with either the leaded gasoline or the control groups, but no statistical changes were found between the groups inhaled the leaded gasoline and the control. The GSH content was lower in both the leaded and unleaded gasoline groups than the control group, but GSH in the unleaded group was higher than in the leaded group. The unleaded gasoline exposed group exhibited a significant decrease in superoxide dismutase activity in comparison with either the leaded gasoline or the control groups. No statistical differences were found between the leaded and the control groups.

**Table 6:** Effect of chronic exposure to two types of gasoline vapour on the contents of lipid peroxidation, reduced glutathione and superoxide dismutase activity of cerebral cortex in male rats.

	GROUP		
**Parameter**	**Control**	**Leaded gasoline**	**Unleaded gasoline**

Lipid peroxidation (MAD) nmol/gwet tissue	4.35 ± 0.79	4.456 ± 0.71	6.57 ± 1.37 a** b**

Reduced glutathione (GSH) mgGSH/g wet tissue	5.834 ± 0.76	3.635 ± 0.43 a***	4.7 ± 0.86 a* b*

Superoxide dismutase (SOD) Unit/g wet tissue	267.26 ± 18.46	253.79 ± 14.74	192.56 ± 19.78 a*** b***

Values are expressed as means ± SD

a: significantly different from the control group.

b: significantly different from the leaded gasoline.

Asterisks indicate the level of significance (* P < 0.05; ** P 0.01; *** P < 0.001.)

The number of animals in each experimental group was 6.

Table 7, 8 shows that the rats exposed to the leaded or the unleaded gasoline expressed a higher number of aggressive events as assessed by tooth chattering, threat posture, leaping and biting as well as boxing position in comparison with those of the control. On the other hand, in the duration of tooth chattering and threat posture were higher than those in the control group. No significant difference was observed between the leaded and unleaded gasoline.

**Table 7:** Effect of chronic exposure to two types of gasoline vapour on the number of aggressive behaviour in male rats.

	Parameter			
**GROUP**	**Number of aggression events**			
	
	**Tooth chattering**	**Threat posture**	**Leaping and biting**	**Boxing position**

Control	1.2 ± 0.33	1.1 ± 0.31	0.9 ± 0.35	0.8 ± 0.25

Leaded gasoline	3.7 ± 0.63 a*	3.9 ± 0.43 a**	3.7 ± 0.58 a**	2.7 ± 0.47 a*

Unleaded gasoline	4 ± 0.87 a*	3.6 ± 0.87 a*	3.4 ± 0.70 a*	3.2 ± 0.74 a**

Values are expressed as means ± SE

a: significantly different from the control group.

Asterisks indicate the level of significance (* P < 0.05; ** P 0.01.)

The number of animals was five pairs per ten days.

**Table 8:** Effect of chronic exposure to two types of gasoline vapour on the duration of aggressive behaviour in male rats.

		Parameter		
	**Duration of aggression/sec**			
	
**GROUP**	**Tooth chattering**	**Threat posture**	**Leaping and biting**	**Boxing position**

Control	0.9 ± 0.23	1.5 ± 0.54	1.9 ± 0.72	1 ± 0.42

Leaded gasoline	4.7 ± 1.1 a**	6 ± 1.2 a*	5.5 ± 1.45	2.2 ± 0.63

Unleaded gasoline	4.4 ± 0.92 a*	6.2 ± 1.31 a*	5.9 ± 1.50	2.2 ± 0.74

Values are expressed as means ± SE

a: significantly different from the control group.

Asterisks indicate the level of significance (* P < 0.05; ** P 0.01.)

The number of animals was five pairs per ten days.

## Discussion

The present study revealed that rats exposed to the unleaded-gasoline had significantly elevated levels of lipid peroxides and a decrease in the superoxide dismustase activity in the brain cortex region in comparison with the control or with the group exposed to the leaded gasoline. Lipid peroxidation is a marker of oxidative stress that facilitates the evaluation of the damage caused by free radicals on membrane lipids [34,35]. On the other hand, GSH was lower in either the leaded gasoline or the unleaded gasoline than the control group. Correlative study by Raza [3] revealed that after gasoline application to skin, a decrease in glutathione concentration, glutathione *S *-transferase activity, and lipid peroxidation was observed in liver and brain. Exposure to toluene, one of the gasoline constituents, both *in vivo *and *in vitro*, leads to reactive oxygen species formation in many tissues including brain tissue [36-38]. The study of Calderón-Guzmán *et al *[39] revealed that the aldehydes resulting from the oxidation of the methyl groups in the aromatic compounds of toluene and cresols play an important role in the cell-damage process due to their lipophilic properties. A similar result indicated that exposure to low concentrations of toluene leads to persistent effects on cognitive, neurological, and brain-structural properties in the rat [40]. On the other hand, the results derived from the present trial indicate that Na^+^, K^+^-ATPase activity and total protein content in the leaded or unleaded gasoline groups were lower than those of the control group. The inhibition of the plasma membrane Na^+^, K^+^-ATPase might have been attributed to a direct effect of the solvents and alcohols in the gasoline on the membrane causing dissolving the its bilayers. The most outstanding are toluene (highly lipophylic) acts on membrane lipids and the effect is specifically on phospholipids and on the other hand the organic solvents act on membrane proteins [34,41,42]. Also as the functional hydroxyl group (OH^-^) moves away from the functional methyl group (CH_3_) attached to the aromatic ring of the solvents, the biogenic amines, lipid peroxidation, and Na^+^, K^+^-ATPase and total ATPase enzyme activity tend to suffer important changes, indicating that the stearic impediment effect and the inductive effect produced at the aromatic ring of the solvents play an important role in the response of biogenic amine metabolism, together with the enzymes and lipid peroxidation of animal brain membranes [39]. Other study by Castilla *et al *[43] reported a reduction of the enzyme Na^+^, K^+^-ATPase in brain membranes when high doses of toluene vapour (30,000 ppm) were administered subchronically to rats, but there was an increased Na^+^, K^+^-ATPase activity during acute exposure. In agreement of this line of reasoning is the study of Gutteridge and Halliwell [44] showing that Na^+^, K^+^-ATPase reduces its activity up to 50% due to oxidative stress. The present findings show that the rats exhibited a decrease in the activity of AChE in the unleaded group in comparison with either the control or the leaded groups. In addition to its function in degrading acetylcholine and modulation of neural function; AChE as a structural protein appears to play an important role in axonal outgrowth [45]. Correlative study by Capo *et al *[46] on ethylene glycol, which is consider one of the main constitutes of gasoline, demonstrated various types of neuronal toxicities after ethylene glycol intoxication. The mechanism could possibly be related to oxidation of cellular macromolecules or to relative reduction in acetylcholinesterase-containing neurons, thus potentiating cholinergic effects. In the present study, no statistical differences were detected among the serotonin levels of the cerebral cortex in the three groups; the control, the leaded and the unleaded. However, in the hypothalamus, hippocampus, cerebellum, the serotonin level was lower in the groups exposed to either type of gasoline as compared to the control group. The serotonin system originates in Raphe nucleus in brain stem and projects with extensive connections to the hypothalamus, cerebellum, and cereberum [47] and plays an important role in regulation of behaviour, mood, appetite, anxiety and sleep [48]. The rats exposed to unleaded gasoline had a significant decrease in the dopamine of the cerebral cortex as compared with the other two groups, but in the hypothalamus and hippocampus dopamine was lower in either the leaded or unleaded group as compared with the control. On the contrary, dopamine was elevated in the cerebellum of leaded exposed group above both the unleaded gasoline and control. The fluctuations in the levels of dopamine in different brain areas may be related to the effects MMT which is an organic manganese (Mn) compound added to unleaded gasoline and the mechanisms of Mn neurotoxicity has focused on the oxidative properties of Mn and its interactions with the dopaminergic system, including biphasic increases and decreases in dopamine levels associated with Mn exposure [49]. Also, Prabhakaran *et al *[50] suggested a new underlying mechanism in which Mn interaction with cellular DA to potentiate ROS activates NF-B as well as iNOS signaling, and all of which are involved in promoting dopaminergic cell death. On the other hand, another gasoline constituent; ethylene glycol was found to target the dopaminergic neurons in the basal ganglia thereby inducing serious intoxication manifested as focal inflammation and necrosis in the basal ganglia areas of the brain and these effects are associated with Parkinsonism [51]. Toluene, another gasoline constituent, was reported to induce perturbation in the nigrostriatal tract responsible for the coordination of voluntary movement [52]. The cell bodies of the dopamine system are located in the substabtia nigra of midbrain and project to the striatum of the basal ganglia to coordinate skeletal muscle contraction, moreover, DA innervations reaches out to the hypothalamus to regulate secretion of TRH and prolactin secretion, and then projects to the brain limbic system to modulate motivations and emotions [47]. So, gasoline induced impairment of the DA system would result in serious impacts on the neural control of voluntary locomotion and would affect several behavioural aspects.

In the present study, the norepinephrine level in the cerebral cortex and hypothalamus of the unleaded group was less than that in the control. Also norepinephrine was reduced in the hippocampus of both unleaded and leaded gasoline groups in comparison with the control. In the cerebellum, norepinephrine level was higher in the leaded group as compared with the control group. It was reported that volatile solvents such as benzene, a main constituent of gasoline fuel, seems to interact with the synthesis and catabolism of catecholamines and serotonin in the brain, which might explain the neurotoxic effects of these solvents [53]. In this regard, deficiencies of serotonin or other monoamine neurotransmitters such as dopamine and norepinephrine are linked with depression [54,55]. In reference to the present results of the behavioural tests, the rats exposed to either the unleaded or the leaded gasoline exhibited a significantly higher rate of anxiety and emotionality in comparison with the control animals. Although information related to the effect of gasoline inhalation on behavioural aspects is extremely scarce, yet, it is possible to interpret these results in the light of the effects of gasoline constituents. Another study demonstrated that lead exposure enhances predatory aggression in the cat and provide experimental support for a causal relationship between lead exposure and aggressive behaviour in humans [56]. This was concomitant with deficiency in serotonin that plays an important role to counteract the aggressive behaviour [57,58]. A correlative study by Perreault *et al *[59] found that aggressive male coral reef fish had lower relative serotonergic activity than less aggressive males, and treatment with the serotonin reuptake inhibitor antidepressant drug flouxotine reduce the territorial aggressive behaviour. It was postulated that serotonin helps counterbalance the tendency of two other major neurotransmitters in the brain dopamine and noradrenaline to encourage overarousal, fear, anger, tension, aggression, violence, obsessive-compulsive actions, overeating, anxiety and sleep disturbances [60].

## Conclusion

The present results revealed that gasoline inhalation induced significant fluctuations in the levels of the monoamine neurotransmitters in the cerebral cortex, hypothalamus, hippocampus and cerebellum. The group exposed to the unleaded gasoline exhibited an increase in lipid peroxidation and a decrease in AChE and superoxide dismutase. The physiological impairments were accompanied with a higher tendency towards aggressive behaviour as a consequence to gasoline inhalation.

## Abbreviations

Na^+^, K^+^-ATPase: total adenosine triphosphatase; SOD: superoxide dismutase; AChE: acetylcholinesterase; GSH: reduced glutathione; TBARS: lipid peroxidation; DA: dopamine; NE: norepinephrine; 5-HT: serotonin; CNS: central nervous system; ROS: reactive oxygen species; MMT: methylcyclopentadienyl manganese tricarbonyl; TAME: Tertiary amyl methyl ether; MTBE: Methyl tertiary butyl ether; ETBE: ethyl tertiary butyl ether; TBA: tertiary butyl alcohol.

## Competing interests

The author declares that she has no competing interests.
